# Safety of titanium dioxide (E171) as a food additive for humans

**DOI:** 10.3389/ftox.2024.1333746

**Published:** 2024-07-19

**Authors:** David B. Warheit

**Affiliations:** Warheit Scientific LLC, Wilmington, DE, United States

**Keywords:** European Food Safety Authority (EFSA), oral toxicity of TiO_2_ particles in rats, one-generation reproductive toxicity study, Health Canada, United States Food and Drug Administration

## Abstract

Titanium dioxide (TiO_2_), also known as E171, is commonly used as a white colorant in food, pharmaceuticals, cosmetics, and toothpaste. However, in May 2021, the European Food Safety Authority (EFSA) expert panel, in evaluating the safety of titanium dioxide (E171) as a food additive, concluded that a concern for genotoxicity could not be ruled out. This occurred several years after EFSA had previously considered titanium dioxide to be safe as a food additive. EFSA based this new interpretation on the results of genotoxicity tests of TiO_2_ nanomaterials. EFSA noted that available data are insufficient to define threshold doses/concentrations of TiO_2_ particles below which genotoxicity will not occur in tissues containing these particles. Here, it is argued that EFSA made a manifest error regarding the safety of titanium dioxide (E171) particles as a food additive for humans. First, the notion of particle size distribution of TiO_2_ particles is explained. Second, the changing opinions from the various EFSA evaluations in 2016, 2018, 2019 vs. 2021 are discussed. Third, the low toxicity of TiO_2_ particles is described in rats exposed by oral gavage and feeding studies in rats and mice. Fourth, the importance of low absorption rates from the gastrointestinal tract vs. circulation in rats and humans but not in mice is identified. Fifth, other international health scientists have weighed in on the EFSA (EFSA J, 2021, 19 (5), 6585) decision and generally disagreed with EFSA’s opinion on the safety of E171 TiO_2_. A common theme voiced by the United Kingdom, Canada, Australia, and New Zealand agencies is that it is inappropriate to compare nanoparticle toxicity studies of dispersed/sonicated nanoparticles with the content of E171 TiO_2_ in foods because the test materials used in key studies considered by EFSA (EFSA J, 2021, 19 (5), 6585) are not representative of E171 TiO_2_ particles. Finally, a group of experts recently considered the genotoxicity of TiO_2_ and could not find support for a direct DNA damaging mechanism of TiO_2_ (nano and other forms). For these reasons, it is suggested that EFSA made a manifest error on the safety of E171 as a food additive.

## Introduction

In May 2021, the European Food Safety Authority (EFSA) expert panel in evaluating the safety of TiO_2_ (E171) as a food additive concluded that a concern for genotoxicity could not be ruled out. This occurred several years after EFSA had previously considered titanium dioxide particles to be safe as a food additive. EFSA based this new interpretation on the results of genotoxicity tests of TiO_2_ nanomaterials. EFSA et al. (2021) presented the results of numerous *in vitro* and *in vivo* genotoxicity tests of TiO_2_ nanomaterials. EFSA noted that available data are insufficient to define threshold doses/concentrations of TiO_2_ particles below which genotoxicity will not occur in tissues containing these particles. The abstract of the EFSA et al. (2021) decision is reproduced in Box 1, as this key document is referred to throughout the present perspective.

In this perspective, the author argues that EFSA made a manifest error regarding the safety of titanium dioxide (E171) particles as a food additive for humans. Food grade titanium dioxide (TiO_2_), also known as E171, is very commonly used as a white colorant in foods, pharmaceuticals, cosmetics, and toothpaste. Other pigmentary TiO_2_ products are utilized in paints, and coatings. E171 is generally an anatase-based mix of pigment-grade based TiO_2_ particles. Here, the notion of particle size distribution of TiO_2_ particles will be explained. Moreover, the toxicological database which clearly demonstrates the low toxicity of TiO_2_ particles in rats exposed by oral gavage and feeding studies is discussed.

The perspective is premised on the following arguments. First, the changing opinions from EFSA itself are addressed—altering their recommendations from 2016, 2018, 2019, 2019, vs. 2021 (EFSA et al., 2018; EFSA et al., 2019; EFSA et al., 2019; EFSA et al., 2021). Second, it is noted that the EFSA et al. (2021) disregarded the fact that the absorption of orally exposed TiO_2_ particles in rats and humans is negligible when compared to experimental oral toxicity studies in mice. Moreover, it is argued that the test materials used in key studies considered by EFSA et al. (2021) are not representative of E171 TiO_2_ particles. Third, weight-of-evidence studies are described, disregarded by EFSA, i.e., acute, subchronic, and one generation reproductive toxicity studies, and chronic (2-year) feeding studies conducted by the National Cancer Institute National Toxicology Program—with an anatase based particle distribution of TiO_2_ particles in rats and mice which had demonstrated no adverse chronic effects after a 2-year oral (feeding exposure). Finally, the opinions and documents of several other national scientific health-based agencies are reviewed which have produced thorough and substantive scientific documents—many of which have considered the EFSA recommendation (2021) and have disagreed fundamentally with the EFSA et al. (2021) opinion.

When considered together, it seems likely that the EFSA et al. (2021) decision was poorly conceived from a scientific viewpoint, and it is noted that E171 has subsequently been banned as a food additive by the European Parliament—evidence of political persuasion vs. scientific integrity.

## General particle characteristics of titanium dioxide and its commercial use

The clear majority of applications for titanium dioxide involve its use as a white pigment in coatings (e.g., paints and plastics). TiO_2_ gains its whiteness from its light scattering properties, due to its high refractive index and absence of intrinsic color and particle size distribution. Pigmentary TiO_2_ is required to scatter visible light to appear white, its desirable particle light distribution is primarily between 200 and 300 nm (Braun, 1997) or roughly half the wavelength of visible light (Warheit and Brown, 2019).

**FIGURE 1:**
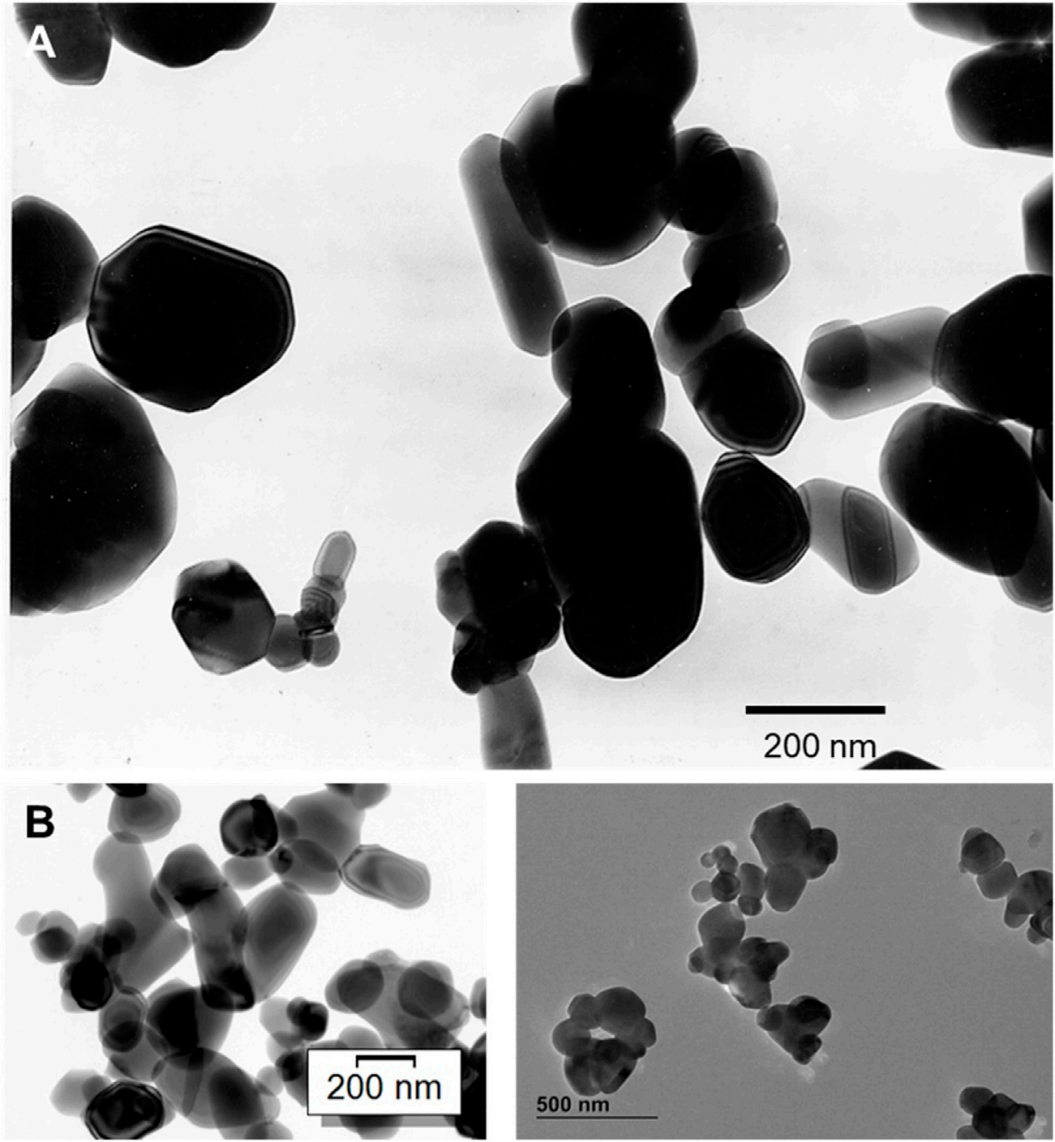
Transmission electron micrographs (TEM) of **(A)** rutile-type, pigment-grade titanium dioxide particles (note the particle distribution of nanoparticles, i.e., < 100 nm and non-nano particles, i.e., > 100 nm), and **(B)** anatase-type, pigment-grade E171 titanium dioxide particles (note the particle distribution of nanoparticles, i.e., < 100 nm and non-nano particles, i.e., > 100 nm).

One of the forms of titanium dioxide (TiO_2_), also known as E171, is very commonly used as a white colorant in foods, pharmaceuticals, cosmetics, and even in toothpaste. In general, E171 is an anatase-based mix of pigment-grade based TiO_2_ particles. However, rutile TiO_2_ is also allowed in the European Union as a food colorant, and there are rutile grades available on the market—even if they admittedly play a minor role. Based upon a particle-size distribution, E171 (like all pigmentary samples has a particle size distribution) is a mixture of TiO_2_ particles which can be defined as non-nano particles (>100 nm) as well as nanoparticles (<100 nm) (Figure 1). However, it is noteworthy that greater than 98% of titanium dioxide (TiO_2_) particles in the commercial market by production volume are of this pigmentary size. Notably, TiO_2_ as an ultrafine or nanoparticulate material, comprises about 2% of global consumption of TiO_2_ and is applied in properties that are distinct from the prescribed pigmentary applications. This is important because TiO_2_ particles of sizes less than 100 nm do not scatter visible light efficiently and are not desired in pigmentary-type applications. Nanoscale TiO_2_, however, has other properties that are useful for applications in catalysis (e.g., automotive catalytic converters), as UV protection agents (e.g., sunscreens) in dye sensitized solar cells or as photocatalysts.

The desired light scattering properties by titanium dioxide (TiO_2_) particulates occur preferentially in the 200 nm–300 nm particle size range (Braun, 1997). Therefore, nanoscale TiO_2_ particles (mean particle sizes <100 nm) are not utilized for these applications. Similar to the TiO_2_ particles used in food-grade applications, a pigment-grade titanium dioxide sample evaluated in a 90-day subchronic oral toxicity study was of a similar particle size range (with a d_50_ of 223 nm by mass and 173 nm by number), containing 21% nanoparticles (defined as < 100 nm). It is important to note that all pigment-grade preparations of TiO_2_ particle-types contain a nano-sized fraction of 20%–30% (as measured by particle number) (Warheit et al., 2015). However, it should be noted that 21% in this case represents a particle number-based value while the nanoscale component represents <1% of the mass of the particle size distribution.

## Oral toxicity studies of TiO_2_ particles in rats according to OECD test guidelines

In a series of *in vivo* oral toxicity studies with a variety of different TiO_2_ particulates, Warheit et al. reported on the results of three OECD test guideline (TG) oral toxicity studies of different durations in rats. Each study was designed to assess different TiO_2_ particles of varying sizes and surface coatings. The results demonstrated an absence of TiO_2_-related toxic effects. In one 90-day subchronic oral toxicity study (OECD TG 408), groups of male and female rats were dosed by oral gavage with rutile-type, surface-coated alumina, pigment-grade test particles (median diameter 125 nm, 21% nanoscale sized particles by particle number criteria) at doses of 0, 100, 300 or 1,000 mg/kg/day. The no-adverse-effect-level (NOAEL) for both male and female rats in this study was 1,000 mg/kg/day—the highest tested level. The NOAEL was determined based upon an absence of adverse effects for any measured in-life, clinical pathology, or anatomic effects. A second study conducted in rats was a 28-day oral toxicity study (OECD TG 407). Young rats were exposed to two uncoated pigment-grade (mean diameter = 173 nm by particle number) to a daily dose of 24,000 mg/kg/day. There were no adverse effects measured during or after the exposure period. The NOAEL for this study was 24,000 mg/kg bw/day. A third acute oral toxicity study (OECD TG 425) involved female rats given a single oral gavage exposure of surface-treated rutile/anatase nanoscale TiO_2_ particle types (mean particle-size = 73 nm by particle number). Doses ranged up to 5,000 mg/kg and evaluated over a 14-day postexposure time period. The oral LD50 dose was determined to be greater than 5,000 mg/kg for this study.

In conclusion, the results from these three studies demonstrated an absence of adverse toxicological effects following oral exposures in rats (Warheit, 2013; Warheit, 2015; Warheit, 2019; Sayes and Warheit, 2009).

Moreover, a study was recently conducted in which the oral bioavailability in male and female CD rats of five different TiO_2_ grades, including E171, was evaluated (Provivo Biosciences, 2022). The study was compliant with Good Laboratory Practice (GLP) and performed in accordance with OECD TG 443 (Extended One-Generation Reproductive Study). The test guideline is designed to provide an evaluation of pre- and postnatal development chemical exposure as well as an evaluation of systemic toxicity in pregnant and lactating females and young and adult offspring. The report described a fully guideline compliant study in which 24 rats/sex/per group were dosed with 0, 100, 300 or 1,000 mg/kg/day of food grade titanium dioxide via the diet before mating and parental females were analyzed for general and reproductive toxicity. The TiO_2_ test materials were administered by single gavage at 1,000 mg/kg bw, and the reference substance was administered orally at 1,000 mg/kg bw. Blood total titanium concentrations were monitored for 96 h post dosing. The investigators concluded that, based upon the results, the NOAELs for systemic toxicity, reproductive toxicity developmental and neurotoxicity were all 1,000 mg/kg bw/day—the highest dose tested for E171 via the diet with no consistent treatment-related effects (Provivo Biosciences, 2022).

The National Cancer Institute (NCI) conducted a 2-year oral bioassay of TiO_2_ particles for possible carcinogenic effects conducted by administering the test chemical in dietary feed to Fischer 344 rats and B6C3F1 mice (National Cancer Institute, 1979). Groups of 50 male and female rats and 50 male and female mice were administered pigment-grade anatase TiO_2_ (Unitane 0–220) (see below) in the diets at one of two doses, i.e., either 25,000 or 50,000 ppm for 103 weeks and subsequently observed for one additional week. Matched controls consisted of 50 untreated rats and 50 untreated mice of each sex. All surviving rats and mice were sacrificed at 104 weeks. The results demonstrated that administration of the TiO_2_ had no appreciable effects on the mean body weights of rats or mice of either sex. With the exception of white feces, there were no other clinical signs that were judged to be related to the oral exposures of titanium dioxide. Survival of male and female rats and the male mice at the end of the bioassay was not affected by the oral exposures or mortality in female mice which was dose related. In the female rats, C-cell adenomas or carcinomas of the thyroid occurred at incidences that were dose related but were not sufficiently high enough for direct comparison of the high-dose group with the control group. Accordingly, the thyroid tumors were not considered to be related to the administration of the TiO_2_ particles. In the male and female mice, no tumors were discovered in dosed groups at incidences that were significantly higher than those for corresponding control groups. The National Cancer Institute (1979) concluded that under the conditions of this bioassay, exposure to TiO_2_ was not carcinogenic by the oral route for Fischer 344 rats or in B6C3F1 mice.

Unitane 0-220 forms of pigmentary anatase TiO_2_ are no longer in production. However, two samples of Unitane 0-220 were retained by member companies of the Titanium Dioxide Manufacturers Association (TDMA), and an unpublished report sent to FSANZ (Food Standards Australia-New Zealand, 2022) provides a comparison of analytical data for these samples, as well as historical records of previous sample analyses, with data on food-grade E171 currently in use (TDMA, 2022). The manufacturer described this substance as anatase with 98% minimum TiO_2_. As described in detail in a report from Health Canada (2022), Unitane 0-220 can be considered comparable to the current form of TiO_2_ as a food additive (E171). These data indicate that Unitane 0–220 has a median particle diameter of 109–135 nm, with 20%–44% of particles by number <100 nm. This is consistent with anatase E171 in samples analyzed by the TDMA. The data also demonstrated that Unitane 0–220 meets the draft EU specification in relation to particle size, absence of surface treatments or coatings and purity including elemental composition and levels of metal contaminants. It is noteworthy that E171 (the subject of the EFSA statement) is a modern-day version of a pigment-grade anatase sample (Weir et al., 2012).

In summary, oral toxicity of TiO_2_ particles is known to be low, in a variety of acute, subchronic, one-generation reproductive toxicity studies—as well as a chronic oral toxicity study.

## Evidence that the absorption of TiO_2_ in the digestive tract is negligible

The alleged potential genotoxicity of E171 in the diet is dependent upon the hypothesis that TiO_2_ particles are taken up in the digestive tract, absorbed into the systemic circulation, accumulate in organs and tissues sufficiently to overload clearance or other homeostatic mechanisms and, thereby, enable genotoxicity. However, there is a significant body of research demonstrating that the systemic absorption of TiO_2_ from the human gastrointestinal tract is negligible at relevant exposure levels. Tissue overload and attendant genotoxicity and other potential adverse effects are not possible without significant absorption from the gastrointestinal tract. EFSA et al. (2021) acknowledged that the absorption of TiO_2_ is very low in the digestive tract but considered the possibility that long-term oral exposures to E171, even at very low rates of absorption, could result in the accumulation of TiO_2_ particles to levels sufficient to pose a potential risk for genotoxicity. However, a thorough review of the literature does not support this assumption (refer to Supporting Information for an evaluation of the relevant key studies available to address this point, i.e., absorption in rats, mice, and humans).

The EFSA et al. (2021) panel calculated estimates of systemic absorption of TiO_2_ particles from the digestive tract by extracting data from several *in vivo* rat studies that the panel selected because they deemed the methods used to detect internal exposures in these studies to be reliable or reliable with some limitations. Although the panel concluded that systemic absorption of orally administered TiO_2_ products is very low, they speculated that long-term accumulation of TiO_2_ in internal organs may lead to genotoxic effects. In the Supporting Information, a review is provided of studies that EFSA et al. (2021) selected or neglected in its assessment, which indicates that there is no credible evidence to support the EFSA et al. (2021) speculation. Overall, *in vivo* studies in rats have demonstrated that systemic absorption of TiO_2_ from single or repeated- oral exposures to TiO_2_ nanomaterials is negligible, even at significant doses orders of magnitude greater than relevant levels of human consumption of E171 used as a food additive, and even when extraordinary procedures are followed to disperse the TiO_2_ particles in aqueous suspensions and to stabilize the suspensions for administration to the animals.

EFSA et al. (2021) summarized many *in vivo* studies on mice in its opinion, including published gastrointestinal absorption and genotoxicity studies. The EFSA et al. (2021) panel estimated that systemic absorption of TiO_2_ in the digestive tract of mice is low. However, studies of mice exposed to high oral doses of TiO_2_ nanomaterials, such as those used in many of the genotoxicity studies that the EFSA et al. (2021) relied upon, suggest that mice have a greater capacity than humans or rats to absorb TiO_2_ from the digestive tract. Overall, the results of studies such as Wang et al. (2007) suggest that mice may have a much greater capacity to absorb TiO_2_ particles in the digestive tract than rats or humans. This observation calls into question the relevance of the results of many of the genotoxicity assays that the EFSA et al. (2021) panel relied upon which tested TiO_2_ nanomaterials in mice.

EFSA et al. (2021) have noted some but not all of the relevant studies of human volunteers, and calculated a worst-case estimate of systemic absorption in the human digestive tract from data extracted from one of these studies. Although the EFSA estimate calculated from these data was very low, again the basis for speculating on the potential accumulation and genotoxicity of TiO_2_ in the internal organs is obscured by the absence of an adequate review of the pertinent studies.

EFSA et al. (2021) acknowledged that the absorption of TiO_2_ is very low in the digestive tract but considered the possibility that long-term oral exposures to E171, even at very low rates of absorption, could result in the accumulation of TiO_2_ particles to levels sufficient to pose a risk for genotoxicity.

Overall, *in vivo* studies in rats and humans have demonstrated that systemic absorption of TiO_2_ from single or repeated oral exposures to TiO_2_ nanomaterials is negligible, even at doses orders of magnitude greater than relevant levels of human intake of E171 used as a food additive.

## Scientific opinions suggesting a disagreement with the EFSA (2021) panel

A number of international health experts from other countries have reviewed the EFSA et al. (2021) opinion and disagreed with the EFSA et al. (2021) position on the safety of E171 TiO_2_ particles as a food additive. Here, the scientific opinions from the scientific experts of 1) United Kingdom, 2) Health Canada, 3) The Food Standards Australia New Zealand (FSANZ), and 4) The US Food and Drug Administration (US FDA) are discussed. In addition, the Titanium Dioxide Manufacturers Association (TDMA) recently commissioned a group of (non-affiliated) genotoxicity experts and asked them to render an opinion on the genotoxicity of E171 TiO_2_ utilized in food applications. The peer-reviewed publication arising from those discussions is also reviewed (Kirkland et al., 2022). In brief, all of the aforementioned groups disagreed with the EFSA et al. (2021) scientific opinion on E171. Only the German Federal Institute for Risk Assessment (BfR) and the French ANSES organization have supported the EFSA et al. (2021) position.

### UK committee on toxicity of chemicals in food, consumer products on the environment

The UK Committee on Toxicity of Chemicals in Food, Consumer Products on the Environment published an interim position paper on titanium dioxide (United Kingdom Committee on Toxicity of Chemicals in Food Consumer Products on the Environment, 2021). The following paragraphs are taken from the report:

“The United Kingdom’s Food Standards Agency reviewed the most recent opinion by EFSA et al. (2021) and have identified a number of concerns. The preliminary findings of these expert committees were published in an interim position statement in January 2022. Upon reviewing the EFSA FAF evaluation with respect to genotoxicity, the COM [Committee on Mutagenicity] concluded that the evidence did not allow definitive conclusion to be drawn and therefore they did not agree with the overall EFSA conclusions on the genotoxicity of E171 titanium dioxide. The COT [Committee on Toxicity] considered that further refinement of data may be needed before making a definitive conclusion of the genotoxicity and safety of TiO_2_, and the conclusions of the EFSA FAF Panel were not justifiable based on the available evidence. Similarly, the COT questioned the quality and robustness of the dataset and the weight given by the EFSA FAF Panel to studies that were considered to be of low reliability”.

“On balance, the Committee considered that the weight of evidence did not support the conclusions drawn by EFSA. The COT also agreed with the comments of the COM with regards to risk communication that as it stands the conclusion is highly risk adverse based on the weak evidence available, and it might create unnecessary concern to the public. They considered that care should be taken when pressing the conclusions as they might cause unnecessary concern and they were uncomfortable with EFSA’s binary communication on a dataset with a lot of uncertainties. They highlighted that the COT does not follow the precautionary approach and reiterated that there is a lot of uncertainty on genotoxicity. The COT suggested that COM should independently review the database on genotoxicity and apply the COM’s guidance on determining thresholds. When considering whether they agreed with EFSA’s conclusion that no differentiation could be made with regards to size/form of titanium dioxide and different aspects of toxicity, the COT erred towards the view that nanoparticles were driving the toxicity. It was decided that an interim position paper, capturing the COT’s view and the proposed next steps should be published”.

### Health Canada—state-of-the-science of titanium dioxide (TiO_2_) as a food additive

In 2022, Health Canada completed a “state-of-the-science” report on titanium dioxide (TiO_2_) as a food additive. The following paragraphs are taken from the executive summary of the report:

“This document was designed to summarize the state of the science regarding the safety of titanium dioxide (TiO_2_) as a food additive. Titanium dioxide has been approved for use as a food additive in Canada over 50 years and is utilized to whiten or brighten many foods. However, recently the safety of titanium dioxide as a food additive has been challenged (EFSA et al., 2021) in the European market, largely as a result of the portion of particles with a diameter less than 100 nm (i.e., nanoparticles) was shown to be as high as 30% on a mass basis. Titanium dioxide particles in the nanoscale, as well as in food-grade TiO_2_ containing nanoparticles are alleged to produce toxic effects in various test systems, when dispersed and stabilized in matrices such as water”.

“Titanium is not metabolized to any significant degree and as a consequence, the vast majority of ingested particles are excreted unchanged in feces. Accordingly, metabolism studies in animals and human volunteers indicate that a small fraction, likely on the order of 0.001% may be systematically available following exposure via the oral route. In the gastrointestinal tract, titanium dioxide particles may gain access to the gut-associated lymphoid tissue (GALT), where they may remain locally in specialized lymphoid follicles known as Peyer’s patches or be translocated systemically, dependent on their size. TiO_2_ has been identified in various organs, notably those rich in macrophages such as liver and spleen, although there is no established link between organ burden and either age or pathology. The initial concerns with human exposure to TiO_2_ particles arose in part from a non-guideline rat study funded by the French Agency for Food, Environmental and Occupational Health & Safety (ANSES) in which animals were exposed to food-grade TiO_2_ dispersed in drinking water at a human relevant dose for 100 days (the Bettini et al., 2017 study). TiO_2_ particles were reported to have accumulated in Peyer’s patches and exposed animals developed large aberrant crypt foci (ACF); a colonic lesion which may progress to neoplasia), at higher rates than unexposed controls. However, the findings of ACF in the colon by Bettini et al. have not been replicated in subsequent studies, even at doses order of magnitude higher. In this regard, Blevins et al. (2019) conducted a study wherein rats received food containing nondispersed E171 for 7 or 100 days. The fact that the particles were not dispersed is representative of normal coloring in foods. The investigators reported that no differences were observed due to E171 in a number of immunological factors, in cytokine production in plasma. No effects on histopathological evaluations of small intestines, liver, spleen, lung or testes. Furthermore, there were no effects on the development of ACFs in the colon. The investigators concluded that dietary E171 administration, even at higher doses produced no effect on the immune parameters or gastrointestinal tissue morphology”.

“Potential toxicity concerns of food-grade TiO_2_ appear to be largely driven by studies that were designed for hazard identification of the material’s constituent particles as opposed to the intact material as encountered in the diet. As dietary studies best reflect how humans are exposed to TiO_2_ in food and given evidence of a significant food matrix effect, the results of dietary studies were accorded the greatest weight in this review. Food-grade TiO_2_, also contains a significant fraction of particles in the nanoscale and therefore, studies conducted with food-grade TiO_2_ will simultaneously evaluate the toxicity of any TiO_2-_NPs that may be present. In addition, GLP- and OECD guideline-compliant studies were deemed the most reliable and of the highest quality; therefore, these studies were provided the highest weight in this review”.

“The summary of the Health Canada document indicated that the putative adverse effects (noted by the EFSA et al. (2021) document, and ANSES-Bettini) were associated with oral exposure to food-grade TiO_2_ largely associated with non-standard studies that utilized the E171 in suspensions of ultrasonically dispersed particles. The opinion of Health Canada’s Food Directorate was that, while these methodologies may be useful for particle characterization—they are not appropriate, and the particle properties do not fully represent exposure to TiO_2_ E171 as a constituent in food applications. Moreover, Health Canada’s Food Directorate did not identify any health concerns for the use of TiO_2_ E171 as a food additive in this course of their review. Furthermore, Health Canada concluded that the weight of evidence suggests, that unlike the decisions of EFSA et al. (2021), a precautionary approach is not warranted at this time”.

### Food standards Australia New Zealand (FSANZ) on titanium dioxide as a food additive

FSANZ issued a report in 2022 on “Titanium Dioxide as a Food Additive”, following the release of the EFSA et al. (2021) assessment. The following paragraphs are taken from the executive summary:

“The Food Standards Australia New Zealand (FSANZ) reviewed the safety of titanium dioxide (E171) when used as a food additive. This assessment was a response to updated evaluation by the European Food Safety Authority (EFSA) which was published in 2021. EFSA concluded that titanium dioxide could no longer be considered safe as a food additive due to a number of uncertainties in recent toxicological studies. This conclusion by EFSA is in contrast to an earlier assessment by EFSA that TiO_2_ is a substance of low toxicity that has been safely utilized as a food additive for many decades”.

“The EFSA 2021 document noted that as particle size distribution of E171 TiO_2_ contains a certain percentage of nanoparticles. Accordingly, FSANZ reviewed the potential health risks associated with oral ingestion of titanium dioxide and other food additives that may contain nanoparticulates. The review in 2016 concluded that there was insufficient data to conclude that there was a health risk associated with consumption of TiO_2_—particularly given the long history of safety and use of this food coloring particulate”.

“The updated review by EFSA et al. (2021) questioning the safety of E171 in foods utilized studies that were performed nearly exclusively with TiO_2_ nanoparticles. EFSA concluded that although the data was not conclusive on TiO_2_ E171, EFSA could not certify that the food additive was safe as a result of the accumulation of nanoparticles in the body concomitant with potential inflammation, neurotoxicity, immunotoxicity—and potential development of aberrant crypt foci—ACF (in the colon), a lack of adequate carcinogenicity studies on TiO_2_ nanoparticulates; and a concern for genotoxicity of TiO_2_ nanoparticles”.

FSANZ noted that despite the ACF finding in the Bettini et al. (2017) study, with sonicated food-grade TiO_2_ at 10 mg/kg bw/day, the results were not confirmed/repeated in two food-grade studies—wherein food-grade TiO_2_ was administered in the diet at much higher doses (i.e., up to 267 or 1,000 mg/kg bw/day (Blevins et al., 2019; Provivo Biosciences, 2022). Moreover, observations of pre-cancerous lesions were also inconsistent with the findings of a National Cancer Institute (NCI) 2-year chronic feeding study with rats and mice (1979). Indeed, in this study the rodents were exposed to diets of up to 50,000 ppm for 2 years without any evidence of tumor formation.

In conclusion, FSANZ reviewed the safety and concluded that there is no evidence to suggest that dietary exposures to food-grade E171 titanium dioxide particles are a concern for human health.

### Where does the US food and drug administration (FDA) stand on this issue?

Although the US FDA has not made an official update since the EFSA et al. (2021) decision, there are a number of clues to the FDA position—as referenced in an article by Elaine Watson in Food Navigator United States (12 December 2022) entitled “FDA doubles down on titanium dioxide safety as CSPI raises concerns”. According to the article, “the FDA has reviewed the scientific opinion from EFSA that prompted the EU ban and takes a different view”. The position that the US FDA has taken is similar to Health Canada, which is that food-grade TiO_2_ is safe for consumption. When queried about whether the US FDA has reconsidered the status of food-grade TiO_2_ following the EC’s decision, an FDA spokesperson indicated that “the FDA reviewed the findings of EFSA et al. (2021) opinion on titanium dioxide and noted that EFSA et al. (2021) opinion continued to confirm no general and organ toxicity, as well as no effects on reproductive and development toxicity.” According to the article, the spokesperson added, “The FDA continues to allow for the safe use of titanium dioxide as a color additive generally according to the specifications and condition, including that the quantity of titanium dioxide does not exceed 1% by weight of the food, found in FDA regulations at 21 CFR 73.575” (Food Navigator USA, 2022).

### Peer-reviewed publication on the genotoxicity assessment of titanium dioxide

The Titanium Dioxide Manufacturers Association (TDMA) set up an independent panel of genotoxicity experts, chaired by Dr. David Kirkland, which performed a comprehensive weight of evidence (WoE) assessment of the genotoxicity of titanium dioxide, based on the available data (Kirkland et al., 2022). In conducting this evaluation, a total of 192 datasets for endpoints and test systems were utilized and were considered the most relevant for identifying mutagenic and carcinogenic potential. These were subsequently reviewed and discussed for both reliability and relevance (by weight of evidence) and in the context of whether the physicochemical properties of the particles had been characterized. The view of an independent panel of experts was that, of the 192 datasets identified, only 34 met the reliability and quality criteria for being most relevant in the evaluation of genotoxicity. Of these datasets, 10 were positive (i.e., reported evidence that titanium dioxide was genotoxic), all of which were from studies of DNA strand breakage (comet assay) or chromosome damage (micronucleus or chromosomal aberration assays). All of the positive findings were associated with high cytotoxicity, oxidative stress, inflammation, apoptosis, necrosis, or combinations of these effects. Considering that DNA and chromosome breakage can be secondary to physiological stress, it is highly likely that the observed genotoxic effects of titanium dioxide, including those with nanoparticles, are secondary to physiological stress. Consistent with this finding, there were no positive results from the *in vitro* and *in vivo* gene mutation studies evaluated, although it should be noted that to definitely conclude a lack of mutagenicity, more robust *in vitro* and *in vivo* gene mutation studies would be useful. Existing evidence does not therefore support a direct DNA damaging mechanism for titanium dioxide (nano and other forms).

With regard to methodology processes, to identify those data sets that were most relevant for study and assessment, the following parameters were assessed: 1) Relevance of the endpoint and test system investigated in terms of their association with genetic or carcinogenic hazard; 2) Reliability of the methods, including characterization of the test substance (in particular for nanoparticles); 3) Quality and interpretation of the reported data by weight of evidence using expert judgment. Comparisons between the EFSA and Expert Panel approaches highlight differences in terms of the types of studies and endpoints that were included or excluded in the respective assessment, how reliability was scored, and how different aspects of test design were assessed. Specifically, Kirkland et al. (2022) question how one expects to observe comet and/or chromosomal aberration assays under conditions where there is cytotoxicity/apoptosis/or necrosis-related nucleases released from lysosomes leading to DNA single/double strand breaks and thus, the positive genotoxicity is secondary, but not a direct event. Furthermore, when dealing with the micronucleus assay (in addition to a positive being secondary to cytotoxicity/apoptosis/necrosis (there is a need to distinguish between micronuclei resulting from chromosomal damage *versus* the compound in question acting as an aneugen (induces numerical chromosome aberration through interactions with cellular targets other than DNA, e.g., interferes with spindle fibers during mitosis). The take-home message from this publication is that it is imperative to incorporate sensitive evaluations of cytotoxicity (e.g., MTT Assay, ATP levels, histopathology (*in vivo*) into the experimental design of genotoxicity assays (Kirkland et al., 2022). Any positive result obtained using levels of a test compound above that which causes cytotoxicity should be deemed a false positive, i.e., a secondary event and not a valid indication of the compound in question being a genotoxic agent.

In conclusion, according to Kirkland et al. (2022), the 34 robust datasets reviewed in the study do not support a direct DNA damaging mechanism for TiO_2_ in either the nanoscale or micro forms. Carefully designed studies of apical endpoints (gene mutation, MN and/or chromosomal aberrations) following OECD recommended methods, performed with well characterized preparations of TiO_2_ particles, could allow firmer conclusions on mutagenicity to be reached.

## Alternative opinions—suggesting agreement with the EFSA (2021) panel

As mentioned above, two European agencies agreed with the EFSA et al. (2021) opinion—the German Federal Institute for Risk Assessment (BfR), and the French Agency for Food, Environment and Occupational Health and Safety (ANSES). The rationale for both of these agencies is described below.

### The German federal institute for risk assessment (BfR)

Bundesinstitut für Risikobewertung (Bfr) (2021) BfR published a statement entitled the “Re-evaluation of titanium dioxide: BfR draws similar conclusions as the European Food Safety Authority” (8 December 2021). According to the BfR, “EFSA concludes that genotoxic effects cannot be ruled out with sufficient certainty. As a food additive, titanium dioxide can therefore no longer be regarded as safe. Since no harmless dose has been determined for genotoxic substance so far, no acceptable daily intake (ADI) could be derived for the substance”. The BfR has dealt with the data on genotoxicity considered by EFSA and mostly arrived at the same conclusions as EFSA. However, the BfR points out that there are still gaps in knowledge for a final assessment. For example, it remains unclear to what extent and in what way titanium dioxide could damage the genetic material.

Notably, with regard to the occurrence of aberrant crypts in exposed animals, the BfR wrote that, “Bettini’s study et al. (2017) was previously assessed by the ANS panel, and the limitations were discussed in detail (EFSA et al., 2018). In the study, there was an increased incidence in adult male Wistar rats—aberrant crypt foci (ACF) at dose of 10 mg/kg body weight per day. Blevins’ study et al. (2019) [0, 40, 400 or 5,000 ppm for 100 days—calculated doses of 1.3, 3.6, 22.4 or 267 mg/kg bw/day] and the newly submitted (unpublished) (OECD TG 443 study) 0, 100, 300 or 1,000 mg/kg bw/day (LPT, 2020)] on reproductive toxicity evaluated by EFSA could not confirm these findings. The EFSA rates the last two studies mentioned as less informative because the exposure of the test animals to titanium dioxide nanoparticles is unclear here”.

### The French agency for food, environmental, and occupational and health safety

The French government agency ANSES developed an opinion on the risk assessment of the nanometric fraction of the food additive E171 (27 October 2022). The title of the document (in French) refers to “the risk assessment of the nanometric fraction of the food additive”, thus giving an indication of the focus on the concern for impacts of nanoparticles contained within pigment-grade E171 titanium dioxide. The long-standing concern for nanomaterials is noted in the Introduction section: “In France, in 2019, the marketing of food products containing TiO_2_ was suspended for 1 year by decree, the DGCCRF being responsible for monitoring the execution of this decree. Effective 1 January 2020, and backed by the precautionary principle, the suspension has, since that date, been renewed each year”. This decision follows a line of mobilizations and the publication of the ANSES opinion of April 2019, recalling the uncertainties regarding the health effects of this food additive. EFSA reacted to this publication, considering that the uncertainties raised by ANSES’s work were already those that EFSA had identified and that the results did not call into question its conclusions as to the safety of this additive (2019). The French decision had numerous consequences in political and economic terms at the European level while the results did not call into question its conclusions as to the safety of this additive (EFSA et al., 2019). It should be noted that ANSES funded the Bettini et al. study (2017). This study required the sonication of the E171 particles, and led to the reported development of ACFs (aberrant crypt foci) (a colonic lesion which may progress to neoplasia) in rats. However, again it must be noted that the findings of Bettini et al. (2017) have not been replicated in subsequent feeding studies—with much higher concentrations of E171 particles (Blevins et al., 2019).

According to the author of this perspective, the latter statement points out the flawed logic in the rationale for both the BfR, ANSES (2022). Bettini et al. (2017) (10 mg/kg bw/day and the testing program that EFSA et al. (2021) and the BfR utilized was based on artefacts by sonicating the E171 preparation to create an artificial preparation of TiO_2_ nanoparticles. Alternatively, the Blevins et al. (2019) study, as well as the extended one generation feeding study, utilized much higher concentrations of E171 and physiologically more relevant) when compared to the Bettini et al. (2017) study. Humans do not sonicate their food before consumption. To sum up this discussion, a common theme of criticisms by the United Kingdom, Health Canada, and FSANZ was that the comparison of nanoparticle genotoxicity studies (sonicated) to E171 TiO_2_ was scientifically inappropriate due in large part to the intended dispersal/sonication of the nanoparticles. In addition, the assessments by Kirkland et al. (2022) (genotoxicity experts) question the validity of the EFSA et al. (2021) panel with regard to genotoxicity assessments.

## Conclusion

In conclusion, this author contends that EFSA made a manifest error regarding the safety of TiO_2_ E171 as a food additive based upon the contention that there was a concern for genotoxicity. First, EFSA changed their opinions from 2016, 2017, 2018, and 2019 vs. 2021, in the absence of new data. In a rebuttal to the EFSA et al. (2021) opinion, the low oral toxicity of and absence of genotoxic effects of TiO_2_ particles in rats is referenced following oral consumption. In addition, the results of a one-generation-reproduction toxicity study is described in rats, and a chronic oral toxicity study conducted by the US National Cancer Institute (NCI) in a 2-year feeding study with rats and mice, the results of which produced no significant toxicity. Furthermore, the functionality of the human gastrointestinal system, and the absorption rates of TiO_2_ following oral exposure intake, differs in rats and humans as compared to mice. Next, the thorough review of the EFSA document (2021) as well as several other national health organizations in the United Kingdom, Health Canada, Australia, and New Zealand, and the United States Food and Drug Administration is noted. These international organizations have disagreed with the conclusions of EFSA et al. (2021) relative to the safety of E171 as a food additive. A common theme of the criticism from the various national health agencies was that EFSA et al. (2021) utilized test materials in key studies that are not representative of E171 particles. An example of this was the utilization of the Bettini et al. (2017) study, wherein the E171 particles were significantly sonicated/dispersed prior to treatment and found to produce aberrant crypt foci, while the Blevins et al. (2019) study utilized significantly higher doses of E171 in rats—and concluded that the feeding study did not produce similar effects as reported by Bettini et al. (2017). In reality, one does not sonicate the food prior to ingestion. Thus, the Bettini et al. (2017) study represents an artefact. Finally, Kirkland et al. (2022), evaluating 34 datasets that were utilized by EFSA et al. (2021), demonstrated that existing evidence does not support a direct DNA damaging mechanism for titanium dioxide—nanoscale forms and pigment-grade forms.

Based upon the data reported herein, it seems reasonable to conclude that EFSA et al. (2021) made a manifest error on the safety assessment of titanium dioxide E171 as a food additive for humans.

BOX 1 **EFSA opinions on titanium dioxide (TiO_2_).**

Timeline of EFSA opinions on titanium dioxide (TiO_2_) (E171) as a food additive:

a. EFSA 2016 opinion: “the use of TiO2 as a food additive does not raise a genotoxic concern”.
b. EFSA et al., 2018 opinion: “did not modify the conclusion on the genotoxicity of TiO2 as stated in the previous EFSA opinion of 2016”.
c. EFSA May 2019 opinion: “ANSES recommends further investigation of *in vivo* toxicity […] this recommendation should be revised once the ongoing work on the physico-chemical characterization of the food additive E71 is completed”.
d. EFSA June 2019 opinion: “the characterization of titanium dioxide (E171) does not provide a reason to revise the conclusion on genotoxicity […] previously drawn by the ANSES panel”.
e. EFSA et al. (2021) opinion: “After conducting a review of all the relevant available scientific evidence, EFSA concluded that a concern for genotoxicity of TiO2 particles cannot be ruled out. Based on this concern, EFSA’s experts no longer consider titanium dioxide safe when used as a food additive. This means that an Acceptable Daily Intake (ADI) cannot be established for E171”.

Abstract of EFSA (2021) opinion on titanium dioxide (E171) as a food additive

The present opinion deals with an updated safety assessment of the food additive titanium dioxide (E 171) based on new relevant scientific evidence considered by the Panel to be reliable, including data obtained with TiO2 nanoparticles (NPs) and data from an extended one-generation reproductive toxicity (EOGRT) study. Less than 50% of constituent particles by number in E171 have a minimum external dimension <100 nm. In addition, the Panel noted that constituent particles <30 nm amounted to less than 1% of particles by number. The Panel therefore considered that studies with TiO2 NPs <30 nm were of limited relevance to the safety assessment of E171. The Panel concluded that although gastrointestinal absorption of TiO2 particles is low, they may accumulate in the body. Studies on general and organ toxicity did not indicate adverse effects with either E171 up to a dose of 1,000 mg/kg body weight (bw) per day or with TiO2 NPs (>30 nm) up to the highest dose tested of 100 mg/kg bw per day. No effects on reproductive and developmental toxicity were observed up to a dose of 1,000 mg E171/kg bw per day, the highest dose tested in the EOGRT study. However, observations of potential immunotoxicity and inflammation with consumption of E171 and potential neurotoxicity with TiO2 NPs, together with the potential induction of aberrant crypt foci with E 171, may indicate adverse effects. With respect to genotoxicity, the Panel concluded that TiO2 particles have the potential to induce DNA strand breaks and chromosomal damage, but not gene mutations. No clear correlation was observed between the physico-chemical properties of TiO2 particles and the outcome of either *in vitro* or *in vivo* genotoxicity assays. A concern for genotoxicity of TiO2 particles that may be present in E 171 could therefore not be ruled out. Several modes of action for the genotoxicity may operate in parallel and the relative contributions of different molecular mechanisms elicited by TiO2 particles are not known. There was uncertainty as to whether a threshold mode of action could be assumed. In addition, a cut-off value for TiO2 particle size with respect to genotoxicity could not be identified. No appropriately designed study was available to investigate the potential carcinogenic effects of TiO2 NPs. Based on all the evidence available, a concern for genotoxicity could not be ruled out, and given the many uncertainties, the Panel concluded that E171 can no longer be considered as safe when used as a food additive.

## Data Availability

The original contributions presented in the study are included in the article/Supplementary Materials, further inquiries can be directed to the corresponding author.
