# Supplementary material for: Safety of titanium dioxide (E171) as a food additive for humans
Source: Front Toxicol. 2024 Jul 19;6:1333746. doi: 10.3389/ftox.2024.1333746 (PMC11295244; doi:10.3389/ftox.2024.1333746)
Supplement: Supplementary file 1 [file DataSheet1.docx]

Supporting Information

**Safety of Titanium Dioxide (E171) as a Food Additive for Humans**

David B. Warheit^1,*^

^1^*Warheit Scientific LLC, Andover Road, Wilmington, DE 19803, USA*

*Correspondence. E-mail: [david.warheit@gmail.com](mailto:david.warheit@gmail.com)

***Systemic gastrointestinal absorption studies of TiO_2_ in rats***

Cho et al. (2013) exposed male and female Sprague-Dawley rats (n=11/group) to 0, 268.4, 529.8, or 1041.1 mg/kg bw/day TiO_2_ nanomaterial suspended in distilled water (DW) by gavage 7 days/week for 90 days. The test material was 80% anatase/20% rutile phase TiO_2_ with a 21 nm and 26.4 ± 6.1 nm nominal and measured number-average primary-particle size (by SEM) and 37.8 ± 0.4 nm hydrodynamic size (dynamic light scattering [DLS]). The control animals received only the distilled water vehicle. Urine and fecal samples were collected for 24 hours from randomly selected animals (n=5/group) that were placed in metabolic cages immediately after administering a dose. The rats were euthanized, and blood, liver, spleen, kidney and brain samples were collected. All samples were analyzed for titanium content by ICP-MS (limit of detection [LOD] = 0.1-1 ng/liter). This study was performed in accordance with OECD TG 408 and GLP guidance of the Korean FDA.

The blood titanium concentrations after 90-days of exposure ranged from 0.4 to 0.5 µg/g across all groups, including the controls, with less than 0.1 µg/g difference between the mean concentration in animals exposed to 1041.5 mg/kg bw/day and the controls. Furthermore, the mean titanium levels ranged from ~0.2 µg/g to 0.8 µg/g in the organs and ~0.4 µg/g to 1.2 µg/g in the urine, with no statistically significant or otherwise discernable differences between the exposed and control animals, and no evidence of a dose-response relationship. In contrast, 24-hour mean titanium fecal concentrations ranged from 0 in controls to ~7500 µg/g in the exposed animals with a clearly evident dose-response relationship.

Cho et al. (2013) also compared the absorption pattern of orally administered titanium dioxide nanoparticles vs. zinc nanoparticles (ZnO) and concluded that ZnO nanoparticles demonstrated much higher absorption rates and concluded from this study that gastrointestinal absorption of TiO_2_ is very low, vs. ZnO – which absorption and thus demonstrated higher absorption and more extensive distribution when administered orally. For the TiO_2_ nanoparticle-exposure, there was extremely low absorption in rats orally exposed for 90 days to doses as high as 1041.5 mg/kg bw/day TiO_2_ nanomaterial, which is 100 to 1000 times greater than relevant human oral exposures to TiO_2._  The investigators concluded that the higher absorption of ZnO compared to TiO_2_ might be due to the higher dissolution rates in acidic gastric fluids.

Janer et al. (2014) administered a single 0 or 100 mg/kg dose of mainly anatase TiO_2_ nanomaterials to male Sprague-Dawley rats (n=6/group) by gavage. The nanomaterial, which was synthesized by Flame Spray Pyrolysis, was suspended (10 mg/ml) in 2 mM aqueous sodium citrate solutions (dispersant) and sonicated for 15 minutes before administering to the animals. The nanomaterial contained spherical particles with a number-average primary-particle size of 18 ± 8 nm (measured by TEM) and hydrodynamic aggregate size of 202 nm in the odium citrate solution.

The rats were euthanized the day after exposure and spleen, liver, small and large intestines, and MLN (mesenteric lymph node) were collected. The intestines were washed with phosphate buffered saline (PBS), Peyer’s patches were excised from the small intestines, and the caecum was excised from the large intestines. One Peyer’s patch and section of the smooth small intestines from each animal was fixed for TEM analysis and the others were prepared for chemical analysis by ICP-MS.

Janer et al. (2014) reported no statistically significant differences in titanium levels in any of the tissues of the exposed rats, when compared with controls, and no nanoparticles were found in the smooth small intestine sections of these animals. However, at least one cell containing TiO_2_ aggregates was observed in one (1) Peyer’s patch of one (1) section from an exposed rat. The aggregates were freely distributed in the cytoplasm, not surrounded by membrane, and not found in the nucleus or mitochondria of the cell. Janer et al. (2014) suggested that this cell may have been a microfold cell (M-cell) that endocytosed a single large TiO_2_ aggregate that was subsequently released to the cytoplasm as smaller aggregates.

These investigators noted that no increases in titanium levels were detected despite selecting tissues that should a priori contain the highest levels of TiO_2_ nanomaterial, and concluded that the oral absorption of TiO_2_ nanoparticles was very low.

Geraets et al. (2014) exposed male Wistar rats (n=3/group) to 2.3 mg/rat/day NM-101, NM-102, NM-103, and females to 2.3 mg/rat/day NM-101 by gavage for 5 consecutive days.

Control males and females received only the ethanol (EtOH)/rat serum albumin (RSA)/PBS vehicle. The test materials were characterized as follows:

- NM-101: anatase TiO_2_ phase; photocatalyst (7-10 nm nominal, 6 nm primary, 38 nm mean particle size);
- NM-102: anatase TiO_2_ phase; photocatalyst (15-25 nm nominal, 20 nm primary, 132 nm mean particle);
- NM-103: rutile (hydrophobic) TiO_2_ phase; cosmetic ingredient (20 nm nominal, 20 nm primary, 186 nm mean particle size);
- NM-104: rutile (hydrophilic) TiO_2_ phase; cosmetic ingredient (20 nm nominal, 20 nm primary, 67 nm mean particle size).

Geraets et al. (2014) reported that the hydrodynamic number-average particle size in the exposure suspensions (measured by DLS) ranged from 80-90 nm for NM-103 and NM-104 to 140-150 nm for NM-101 and NM-102. NM-101 and NM-102 also contained a mode of agglomerates between 250 and 2500 nm (unlike NM-103 and NM-104).

The animals were euthanized, and liver, spleen and MLN samples were collected 24 hours after the last dose and analyzed for titanium content by high-resolution ICP-MS (hr-ICP-MS).

The titanium level was 0.08 µg/g in the liver of one male exposed to NM-103 and was at the 0.03 µg/g LOD in one male exposed to NM-102. The titanium level was at the LOD in liver and spleen of one female control animal. All 38 other titanium measurements in the liver and spleen of the exposed and control animals were below the LOD.

In contrast, all mesenteric lymph node samples (MLN samples) contained detectable levels of titanium measured as µg/g or as µg/whole MLN (lowest mean concentrations 0.07 µg/g; 0.11 µg/whole MLN). However, except for NM-104, there was no clear distinction of discernable absorption between the titanium levels measured in the exposed animals compared with the background levels measured in the control animals (0.14 µg/whole MLN). Even for NM-104, the worst-case absorption calculated from the maximum titanium level of 0.36 µg/whole MLN represented only approximately 0.003% of the administered dose of titanium.

The results of the Geraets et al. (2014) study demonstrated that systemic absorption of TiO_2_ after repeated oral exposures to the tested nanomaterials was very low, despite efforts to disperse and stabilize the dispersed nanoparticles before administration to the rats.

MacNicoll et al. (2015) investigated the potential absorption of 4 commercially available TiO_2_ products in male rats (n=6/group) that were bred from parental animals fed a “titanium-free” diet to establish low titanium background exposure. The test materials included (number-average primary particle size):

- #1 - Anatase TiO_2_ (~15 nm nominal; 40 nm measured);
- #2 - Rutile TiO_2_ (<100 nm nominal; 40-50 nm measured);
- #3- Anatase TiO_2_ (~15 nm nominal; 120 nm measured);
- #4 - Rutile TiO_2_ (<5000 nm nominal; up to 5000 nm measured).

Test materials 1 and 2 were dispersed in water or in 5% aqueous ovalbumin, sonicated, and centrifuged to separate nano-fractions from the larger particles. Test material 3 was dispersed in water or in 5% aqueous ovalbumin and centrifuged to isolate the nano-fraction, which was then freeze dried and resuspended, whereupon the particles re-agglomerated to form ~120-nm clusters. Test material 4, which served as the “non-nano control,” was simply dispersed in water with sonication but without ovalbumin or centrifugation.

Each test material was administered as a single 4.6 mg/kg bw dose of TiO_2_ by gavage, and blood, urine and fecal samples were collected 0-2, 24, 48, 72, and 96 hours postexposure. The rats were then euthanized, and liver, brain, heart, kidney, spleen and small and large intestines were collected for analysis of titanium content by ICP-MS.

These investigators reported that there were no statistically significant differences in the levels of titanium measured in the blood, urine, or internal organs and tissues of the animals exposed to any of the test materials at any time, compared with the corresponding levels measured in control animals fed the titanium-free diet without TiO_2_ added. The levels of titanium in the organs of the exposed rats appeared to be close to or below the LOD. In the gastrointestinal tract samples, titanium levels were statistically significantly elevated only in rats exposed to test material 2, compared with the controls. The TiO_2_ administered was found to be substantially excreted in the feces in all cases.

MacNicoll and coworkers concluded that no appreciable amounts of any of the TiO_2_ of the test materials were systemically absorbed from the digestive tract in this study.

Kreyling et al. (2017) exposed female non-fasted Wistar-Kyoto rats (n=4/group) to a single 30 to 80 µg/kg bw dose of ^48^V-TiO_2_ (anatase) radiolabeled nanomaterial (70-nm median hydrodynamic diameter) by gavage. The rats were then held individually in metabolism cages to collect urine and feces. The animals were exsanguinated 1 hour, 4 hours, 24 hours or 7 days postexposure, and blood, liver, spleen, kidneys, lungs, heart, brain, and uterus were collected. ^48^V-radioactivity was measured in the excreta, blood, organs, and carcass by ϒ-ray spectrometry, and TiO_2_ content of each toxicokinetic compartment at each postexposure time was estimated from the measurements.

The test materials were prepared by irradiating samples of TiO_2_ nanomaterial with a proton beam to yield a 1.0 MBq/mg ^48^V-TiO_2_ preparation, which was administered to rats euthanized 1 hour, 4 hours, 24 hours postexposure, and a 2.35 MBq/mg ^48^V-TiO_2_ preparation, which was administered to rats euthanized 7 days postexposure. These preparations were washed repeatedly to remove ^48^V ions before administering to the animals.

Kreyling et al. (2017) made corrections to the organ- and tissue- ϒ-ray measurements based on the assumption that only 60% to 70% of the blood volume was recovered by exsanguination.

The authors reported that 77.4% ± 3.1% and 99.7% ± 0.1% of the dose was excreted in the feces 24 hours and 7 days after exposure, respectively. They estimated, from the radioactivity measured 7 days after exposure, the following ^48^V-TiO_2_ organ/tissue levels:

- liver 0.09 ng/g
- lungs 0.10 ng/g
- kidneys 0.29 ng/g
- brain 0.36 ng/g
- spleen 0.45 ng/g
- uterus 0.55 ng/g
- skeleton 0.98 ng/g

Kreyling et al. (2017) concluded that the absorbed fraction of ^48^V-TiO_2_ across the intestinal epithelium was very low and the absorbed fractions in the liver, spleen and other organs were even lower by an order of magnitude.

Donner et al. (2016) reported the results of *in vivo* bone marrow micronucleus (MN) tests performed by 2 different laboratories in which Sprague-Dawley or Wistar rats (n=5=7/sex/group) received a single 0, 500, 1000, or 2000 mg/kg bw dose of one of 3 pigment-grade TiO_2_ products or one of 3 TiO_2_ nanomaterials by gavage. The micronucleus (MN) tests were performed in accordance with OECD TG 475, US EPA OPPTS 870.5395, EC Directive 440/2008/EC Method B.12, and good laboratory practice (GLP) regulations. Approximately 48 and 72 hours after exposure, blood samples were collected and 20,000 peripheral blood reticulocytes (RET) per animal were analyzed. For each of the six tests, there were no biologically or toxicologically relevant increases in the micronucleated RET frequency in any TiO_2-_exposed group at either time point at any dose level. All six TiO_2_ test substances were negative for in vivo genotoxicity effects; however, it is noted that the exposure to target tissues was likely negligible. One pigment grade and one nanoscale material each were evaluated for potential systemic exposure/uptake from the gastrointestinal tract by analysis of TiO_2_ in blood and liver. No significant increases in TiO_2_ over controls were measured in blood (48 or 72 hrs) or liver (72 hours) following exposure to 2000 mg/kg bw TiO_2_. These data indicated that there was no absorption of the test material from the gastrointestinal tract into the blood circulation and the lack of genotoxic effects is therefore attributed to a lack of exposure due to the inability of the test materials to migrate from the gastrointestinal tract into the bloodstream and then into target tissues.

Overall, *in vivo* studies in rats have demonstrated that systemic absorption of TiO_2_ from single or repeated- oral exposures to TiO_2_ nanomaterials is negligible, even at significant doses orders of magnitude greater than relevant levels of human consumption of E171 used as a food additive, and even when extraordinary procedures are followed to disperse the TiO_2_ particles

in aqueous suspensions and to stabilize the suspensions for administration to the animals.

***Systemic absorption studies of TiO_2_ in mice***

Wang et al. (2007) administered a single 5000 mg/kg bw/day dose of one of 3 TiO_2_ products to CD-1 (ICR) mice (n=10/sex/group) by gavage, following OECD TG 420 (Acute Oral Toxicity – Fixed Dose Procedure). The test materials included 2 nanomaterials (ultrafine; columnar/spindular particles) with average primary-particle sizes of 25 nm and 80 nm and a non-nanomaterial (fine; spherical particles) with average primary-particle sizes of 155 nm, as determined by TEM. These materials were suspended in 0.5% aqueous hydroxypropyl methylcellulose (HPMC), sonicated for 15 to 20 minutes, and then mechanically vibrated for 2 to 3 minutes before administering to the mice. Control mice received the 0.5% HPMC vehicle only. Samples of the blood, heart, liver, spleen, kidneys, lung, brain, testes and ovaries were collected 2 weeks postexposure for analysis of titanium levels by ICP-MS.

Titanium levels were elevated in the liver of the animals exposed to the 25-nm or 80-nm nanomaterial and in the liver of the animals exposed to the 80 nm nanomaterial. In contrast, the titanium levels in the liver and kidneys of the animals exposed to the 155-nm non-nanomaterial were indistinguishable from controls. No effects on blood titanium levels were observed in any of the exposed animals (25-nm, 80-nm, or 155-nm) compared with the controls.

However, Wang et al. (2007) reported increased titanium levels in the spleen and brain of mice exposed to the 155-nm non-nanomaterial, in the liver, spleen, kidneys, lungs, and brain of the mice exposed to the 80-nm nanomaterial, and in the spleen, kidneys, and brain of the mice exposed to the 25-nm nanomaterial.

Wang et al. (2007) administered a dose of each test material (i.e. 5000 mg/kg bw/day) that is 500 to 5000 times greater than relevant human oral exposures to TiO_2_. The results reported in this study can be attributed to particle overloading of the gut and attendant facilitation of the systemic absorption of the particles in the gut. It is possible, too, that the results are attributable to a greater potential for TiO_2_ particle absorption from the digestive tract of mice compared with rats and humans. In any case, the Wang et al. (2007) study does not reflect the potential for absorption in the human gut at relevant levels of human oral exposures.

Titanium Content analysis

Liver

Control ~ 89 ng/g

25 nm 106.31 ng/g

80 nm 3970.4 ng/g

Fine 106.7 ng/g

Kidney

Control ~ 150 ng/g

25 nm ~375 ng/g

80 nm ~420 ng/g

Fine ~170 ng/g

Brain

Control ~ 89 ng/g

25 nm ~106.3 ng/g

80 nm ~970 ng/g

Fine ~106 ng/g

Overall, the results of studies such as Wang et al. (2007) suggest that mice may have a much greater capacity to absorb TiO_2_ particles in the digestive tract than rats or human subjects. This observation calls into question the relevance of the results of many of the genotoxicity assays that the EFSA (2021) relied upon, which tested TiO_2_ nanomaterials in mice.

Ali et al. (2019) evaluated the toxicity of titanium dioxide nanoparticles (TiO_2_NPs) in mice according to their doses and particle sizes. Mice were exposed by oral administration for 5 days to TiO_2_ NPs (21 and 80 nm) with different doses (50, 250 and 500 mg/kg body weight) and were evaluated using measurements of oxidative stress markers; glutathione (GSH), superoxide dismutase (SOD), catalase (CAT), malondialdehyde (MDA) and nitric oxide (NO), liver function indices; aspartate and alanine aminotransferases (AST and ALT), chromosomal aberrations and liver histopathological patterns. The results revealed significant alterations in all the measured parameters and demonstrated positive correlations with the gradual dose increment. In addition, the smaller particle size exposures of TiO_2_NPs (21 nm) had greater adverse effects in all the selected biochemical parameters, genetic aberrations and histological investigations. Accordingly, it was concluded that the toxicity of TiO_2_NPs increases in a dose-dependent manner and concomitant with the impacts correlated with the reduced particle size (i.e., P25 – 21 nm). The evaluated biomarkers were viewed as good indicators for TiO_2_NPs toxicity following oral exposure in mice.

Ali et al. (2019) assessed 5 days of oral exposure [TiO2 NPs 21 and 80 nm) with different doses (50, 250 and 500 mg/kg bw) in mice via measurement of oxidative stress markers – glutathione (GSH) superoxide dismutase (SOD) catalase (CAT) malondialdehydes (MDA), and nitric oxide (NO); liver function indices; aspartate and alanine aminotransferases (AST and ALT), chromosomal aberrations and liver histopathological patterns.

Administration of 80 nm TiO2-NPs recorded cytogenetic disorders in the mice bone marrow represented by increasing the number of aberrant cells reached to 12, 15, and 24% at doses of 50, 250 and 500 mg of TiO2/NPs/kg body weight respectively, and 24% of doses at doses of 50, 250 and 500 mg of TiO2 NPs/kg body weight. The results revealed drastic alterations in all of the measured parameters and showed positive correlations with the gradual dose increment. In addition, the smaller particle size of TiO_2_NPs (21 nm) had more adverse effects in all of the selected biochemical parameters, genetic aberrations and histological investigations.

Based on the findings of Ali et al. (2019), it could be concluded that the risk of TiO_2_NPs via oral exposure may be manifested by increase of ROS, oxidative severe liver damage and genetic alterations. These disturbances are inversely correlated with the particle size and directly correlated with the administrative dose.

Comera et al. (2020) studied jejunal villus absorption and paracellular tight junction permeability as potential major routes for early intestinal uptake of food-grade TiO_2_ particles: an *in vivo* and *ex vivo* study in mice. The investigators studied *in vivo* the gut absorption kinetics of TiO_2_ in fasted mice orally given a single dose (40 mg/kg) to assess the ability of intestinal apical surfaces to absorb particles when available without entrapment in the bolus. The epithelial translocation pathways were also identified ex vivo using intestinal loops in anesthetized mice.

**These investigators reported that** after a single E171 dose in mice, early intestinal uptake of TiO_2_ particles mainly occurred through the villi of the small intestine, which, in contrast to the Peyer’s Patches, represents the main absorption surface in the small intestine.

Overall, the results of studies such as Comera et al. (2020), Ali et al. (2019), and Wang et al. (2007) demonstrate that mice are likely to have an augmented capacity to absorb TiO_2_ particles in the digestive tract when compared to rats or human subjects. This observation calls into question the issue of species differences concomitant with the relevance of the results of many of the genotoxicity assays that the EFSA (2021) relied upon which tested TiO_2_ nanomaterials in mice.

***Systemic absorption studies of TiO_2_ in human subjects***

Böckmann et al. (2000) measured blood titanium levels in volunteers before and after the oral administration of 23 mg or 46 mg TiO_2_ (primary particle size range 160 nm to 380 nm) in gelatin capsules. They reported increases in blood titanium levels with 3 distinct maxima and substantial variations in the postexposure peak-level time, measured by ICP-AES.

However, as Jones et al. (2015) noted, the blood titanium levels did not double when the dose was doubled, contrary to expectations. Furthermore, the pre-exposure and postexposure blood titanium levels reported by Böckmann et al. (2000) were comparable to those measured by Jones et al. (2015), even though the doses tested by Jones et al. (2015) (i.e., equivalent to 300 mg/day for a 60-kg individual) was approximately an order of magnitude greater than the doses tested by Böckmann et al. (2000). These observations seem to be reasonable.

Pele et al. (2015) administered a single 100-mg dose of E171 (260-nm nominal median particle diameter) in two 50-mg gelatin capsules with 250 ml water to each of 8 fasted volunteers. Blood samples were collected from 7 of the subjects (blood could not be drawn from the cannula of 1 subject) 0, 0.5, 1, 1.5, 2, 3, 6, 8 and 10 hours post-exposure. The sample from 5 of the subjects (blood from 2 of the subjects clotted) were examined by dark field microscopy to score for reflectance intensity (grades: 0, 1, 2, and 3), which was attributed to the presence of bright white punctate particles in the samples, and analyzed by hr-ICP-MS to measure the titanium content.

The reflectance scores increased from grade 0 at time 0 (n=5) to peak at a maximum of grade 3 at 6 hours post exposure (n=2) and decreased to grade 2 (n=2) or grade 1 (n=2) within 8-10 hours post exposure. The corresponding mean titanium concentrations (n=5) in the blood samples were very low, increasing from approximately 2 ppb at time 0-3 hours to peak at 12 ppb 6 hours postexposure and then decreased to 5 ppb 10 hours postexposure. The authors stated that the reflectance scores roughly mirrored the titanium concentrations measured in the blood samples.

Pele et al. (2015) noted that the ingestion of 100 mg of E171 in gelatin capsules results in intestinal exposure that exceeds, in a single dose, the normal upper limits of intestinal exposures to TiO_2_ from the diet. This observation applies as well to the study by Böckmann et al. (2000), in which volunteers received a single dose of 23 mg or 46 mg TiO_2_ in gelatin capsules. Furthermore, the encapsulation of TiO_2_ very likely interfered with the progressive increase in the agglomeration of TiO_2_ and the attendant decrease expected in potential absorption with the passage of TiO_2_ through the digestive tract, as demonstrated in simulated digestion experiments.

Jones et al. (2015) investigated the potential absorption of 3 commercially available TiO_2_ materials in volunteers (n=9; 4 men, 5 women; 30–56 years of age). Each volunteer received a single 5 mg/kg bw oral dose of a TiO_2_ test material dispersed in water in each of 3 studies. The dose was selected to represent relevant high-end human oral exposures to TiO_2_.

The test materials included products with nominal average TiO_2_ particle size 15 nm (study 1), 100 nm (study 2), and <5000 nm (study 3), with 4 to 16 weeks between each study and most of the volunteers participating in all three studies. The authors characterized each test material by centrifugal particle sedimentation (CPS) disc centrifuge analysis to reveal that the test materials were broadly consistent with the manufacturers’ specifications:

- Study 1: 100% anatase TiO_2_ (peak particle size 10 nm, ~100% of particles <50 nm by number, and ~30% <50 nm by weight);
- Study 2: 95% rutile TiO_2_ (peak particle size 70 nm, 95% of particles within the 48 nm and 154 nm range by number);
- Study 3: 100% rutile TiO_2_ (peak particle size 1800 nm, 100% of particles >105 nm by number, and ~100% >700 nm by mass).

Analysis by TEM showed that all of the test materials were substantially agglomerated (up to 1000 nm), although the individual particles were readily distinguishable.

Jones et al. (2015) collected urine from the subjects for 24 hours before and 72-hours after exposure and blood samples before and 2, 4, 24 and 48 hours after exposure for analysis for titanium content by ICP–MS.

None of the test materials yielded urine- or blood-concentration absorption/elimination curves that would be expected if TiO_2_ was absorbed in any significant amount in the digestive tract. Moreover, there were no statistically significant differences between mean preexposure and mean postexposure titanium levels in urine, whole blood, erythrocytes or serum when the data were fitted to two sets of multilevel statistical models.

The models suggested statistically significant greater mean pre-dose and post-dose titanium levels in urine and erythrocytes, but not in whole blood or serum, in Study 2 and Study 3, compared with the corresponding values obtained in Study 1. However, the modelled confidence intervals for all media, except erythrocytes, overlapped across Studies 1, 2 and 3, and no statistically significant increases in measured erythrocyte titanium levels or evidence of increased urinary excretion was observed during the post-exposure period.

Jones et al. (2015) reported that no more than 0.1%, if any, of the administered dose of TiO_2_ was absorbed in the gastrointestinal tract of the volunteers, regardless of the differences in particle size distribution and other properties of the materials tested. Furthermore, the lowest dose administered was 315 mg/kg bw TiO_2_ in Studies 1 through 3. The authors noted that 0.1% absorption of 315 mg (i.e., 315 µg) TiO_2_ would be readily detected as a demonstrable elevation in blood titanium levels above background levels.

The clinical parameters monitored, including full blood count (FBC) and liver function test (LFT) measurements, remained within the normal ranges throughout the study.

The authors’ conclusion that the results provided no evidence of significant TiO_2_ absorption in the gastrointestinal tract regardless of the particle size distribution of the test material and seems to be an accurate reflection of their study results.

Overall, the results of the studies reported by Böckmann et al. (2000), Pele et al. (2015) and Jones et al. (2015) indicate that the systemic absorption of orally administered TiO_2_ is very low, at most, in the human gastrointestinal tract, regardless of the TiO_2_ product tested. In any case, the results indicate that the potential absorption of TiO_2_ particles in human subjects is much lower than would be predicted by standard nanomaterial-characterization protocols in which the test materials are vigorously dispersed in water or saline solutions and the dispersions are stabilized. This is attributable to the tendency of TiO_2_ particles to form very large particle agglomerations in foods and in the digestive tract.

Thoree et al. (2008) found the pigment mainly in mature macrophages in Peyer’s patches of human subjects, which exhibit low metabolic and immunological activity. These investigators reported that there was no evidence of differential cell phenotype or activation associated with pigment cells in Crohn’s colitis and non-Crohn’s colitis patients. Similarly, no phenotypic changes or activations were observed in normal ileal tissue at the resection margins of bowel removed from adenocarcinoma patients, except for a slight increase in S100 protein expression (common in many human cancers). The authors concluded that the pigment cells are probably inert storage sites for some of the particles that traverse the Peyer’s patches and that there is no evidence of different pigment cell phenotype or activation in differing disease states.

Barreto da Silva et al. (2020) explained that there is no evidence to suggest that pigment cells signal pro-inflammatory or any other adverse effects. M-cells (i.e. microfold-cells) can take up, optimally, large nanoparticles (~50-100 nm) and small microparticles (~100-200 nm). M-cells are specialized intestinal cells of organized lymphoid follicles overlying Peyer’s patches in the small intestines. These cells actively sample or phagocytose microorganisms and transport them across the epithelial surface for uptake and processing by dendritic cells (DCs). M-cells are normally responsible for taking up calcium phosphate particles and associated soluble macromolecules so that particle-scavenging immune cells in the Peyer’s patches can recognize antigens among the macromolecules and, thus, maintain immune-system tolerance (i.e. unresponsiveness) or trigger an immune response, as appropriate. A substantial fraction of the typical particle-size distribution of E171 falls within the optimal range for uptake by M-cells.

Heringa et al. (2018) analyzed the formalin-fixed livers and spleens of deceased men (n=6, 56-87 years of age) and women (n=9, 77-104 years of age) for titanium concentration by hr-ICP-MS and for the presence of TiO_2_ nanoparticles by sp-ICP-MS. All of the subjects had lived in the Netherlands their entire lives and were assumed to have followed a Dutch diet. The liver and spleen samples with the highest levels of particles measured by ICP-MS were analyzed by SEM energy dispersive X-ray detection (SEM-EDX) to confirm the results.

For hr-ICP-MS analysis, the tissues were ground, homogenized, and digested in mineral acids in a microwave oven system. For sp-ICP-MS analyses, the fixed tissue samples were depolymerized in digestion buffer for 3 hours at 100°C, cooled, digested enzymatically with proteinase K at 37°C for 16 hours, and homogenized by sonication for 1 minute before instrumental analysis. Samples of fixed liver and spleen were simply ground for SEM-EDX analysis.

The results reported in this study can be summarized as follows:

- Titanium concentrations were below the LOD (10 ppb) in the liver samples of 8 subjects and no particles were found in the liver samples of 8 subjects.
- Particles with average size ranging from 86 to 421 nm were found in the liver samples of 7 subjects.

Heringa et al. (2018) reported an average liver titanium concentration of 40 ± 20 ppb for all subjects combined, assuming ½LOD for subjects in which no titanium was detected.

Titanium concentrations were below the LOD in the spleen sample of 1 subject and no particles were found in the spleen samples of 2 subjects. Particles with average size ranging from 88 to 445 nm were found in the spleen of 13 subjects. The authors reported an average spleen titanium concentration of 80 ± 100 ppb for all subjects combined, again assuming ½LOD for subjects in which no titanium was detected.

The results and interpretation of the Heringa et al. (2018) study may be open to confounding by even the smallest contamination, given the tissues were extracted during autopsies, preserved and stored in formalin solution for unspecified durations, and then subjected to the grinding, homogenization, digestion, and other steps of the complex tissue-processing protocol.

Nevertheless, EFSA (2021) estimated that the oral systemic bioavailability of E171 is less than 1%, at most, based on the sum of the average Ti levels in the liver and spleen calculated only from measurements greater than the LOD in the study by Heringa et al. (2018). EFSA (2021) concluded that the levels of TiO_2_ measured in the tissues reflected steady state levels, given the ages of the subjects, and that the absorption of TiO_2_ used as a food additive is low under normal life conditions. This conclusion seems to be accurate, which is consistent with the long history of use of E171 as a food ingredient with no reports of adverse health effects.

The purported potential genotoxicity of E171 in the diet depends on the hypotheses that TiO_2_ particles are taken up in the digestive tract, absorbed into the systemic circulation, accumulate in organs and tissues sufficiently to overload clearance or other homeostatic mechanisms and, thereby, enable genotoxicity. However, there is a significant body of research demonstrating that the systemic absorption of TiO_2_ from the human gastrointestinal tract is negligible at relevant exposure levels. Tissue overload and attendant genotoxicity and other potential adverse effects are not possible without significant systemic absorption.

Overall, *in vivo* studies in rats and humans have demonstrated that systemic absorption of TiO_2_ from single or repeated oral exposures to TiO_2_ nanomaterials is negligible, even at doses orders of magnitude greater than relevant levels of human intakes of E171 used as a food additive, and even when extraordinary procedures are followed to disperse the TiO_2_ particles in aqueous suspensions and to stabilize the suspensions for administration to the animals.

**References**

Ali SA, Rizk MZ, Hamed MA, Aboul-Ela. El-Rigal NS, El-Rigal NS, Aly HF, Abdel-Hamid Z. Assessment of titanium dioxide nanoparticles toxicity via oral exposure in mice: effect of dose and particle size. Biomarkers. 2019;24(5):492-498.

Barreto da Silva A, Miniter M, Thom W, Hewitt RE, Wills J, Jugdaohsingh R, Powell JJ. Gastrointestinal absorption and toxicity of nanoparticles and microparticles: myth, reality and pitfalls explored through titanium dioxide. Curr Opin Toxicol. 2020;19:112-120.

Böckmann J, Lahl H, Eckert T, Unterhalt B [Blood titanium levels before and after oral administration titanium dioxide] [German]. Clinical Trial Pharmazie 2000. 55:140-143.

Cho WS, Kang B-C, Lee JK, Jeong J, Che J-H, Seok SH. Comparative absorption, distribution, and excretion of titanium dioxide and zinc oxide nanoparticles after repeated oral administration Part Fibre Toxicol. 2013;10:9.

Comera C, Cartier C, Gaultier E, Catrice O, Panouille Q, El Hamdi S, Tirez K, Nelissen I, Theodorou V, Houdeau E. Jejunal villus absorption and paracellular tight junction permeability are major routes for early intestinal uptake of food-grade TiO_2_ particles: an in vivo and ex vivo study in mice. Part Fibre Toxicol. 2020;17(1):26.

Donner EM, Myhre A, Brown SC, Boatman R, Warheit DB. In vivo micronucleus studies with 6 titanium dioxide materials (3 pigment-grade & 3 nanoscale) in orally exposed rats. Reg. Toxicol. Pharmacol. 2016;74:64-74.

Geraets L, Oomen AG, Krystek P, Jacobsen NR, Wallin H, Laurentie M, Verharen HW, Brandon EFA, de Jong WH. Tissue distribution and elimination after oral and intravenous administration of different titanium dioxide nanoparticles in rats. Part Fibre Toxicol. 2014;11:30.

Heringa MB, Peters RJB, Bleys RLAW, van der Lee MK, Tromp PC, van Kesteren PCE, van Eijkeren JCH, Undas AK, Oomen AG, Bouwmeester H. [Detection of titanium particles in human liver and spleen and possible health implications.](https://pubmed-ncbi-nlm-nih-gov.proxy.kib.ki.se/29642936/) Part Fibre Toxicol. 2018;15(1):15.

Janer G, Mas del Molino E, Fernández-Rosas E, Fernández A, Vázquez-Campos S. Cell uptake and oral absorption of titanium dioxide nanoparticles. Toxicol Lett. 2014; 228: 103-110.

Jones K, Mortona J, Smitha I, Jurkschat K, Hardinga A-H, Evans G. Human in vivo and in vitro studies on gastrointestinal absorption of titanium dioxide nanoparticles. Toxicol. Lett. 2015;233: 95-101.

Kreyling WG, Holzwarth U, Schleh C, Kozempel J, Wenk A, Haberl N, Haberl S, Schäffler M, Lipka L, Semmler-Behnke M, Gibson N. Quantitative biokinetics of titanium dioxide nanoparticles after oral application in rats. Nanotoxicology. 2017;11:443-453.

# MacNicoll A, Kelly M, Aksoy H, Kramer E, Bouwmeester H, Chaundry Q. A study of the uptake and biodistribution. nano-titanium dioxide using in vitro and in vivo models of oral intake. J Nanopart Res. 2015;17:66

Pele LC, Thoree V, Bruggraber SF, Koller D, Thompson RP, Lomer MC, Powell JJ. [Pharmaceutical/food grade titanium dioxide particles are absorbed into the bloodstream of human volunteers.](https://pubmed.ncbi.nlm.nih.gov/26330118/) Part Fibre Toxicol. 2015;12:26.

Thoree V, Skepper J, Deere H, Pele LC, Thompson RPH, Powell JJ. Phenotype of exogenous microparticle-containing pigment cells of the human Peyer’s patch in inflamed and normal ileum. Inflammation Res. 2008;57:374-378.

Wang T, Ma Y, Jia G, Gao Y, Lia B, Sun J, Li Y, Jiao F, Zhao Y, Chai Z. Acute toxicity and biodistribution of different sized titanium dioxide particles in mice after oral administration. Toxicol Lett. 2007;168: 176-185.
